# Trends and variation in antidepressant prescribing in English primary care: a retrospective longitudinal study

**DOI:** 10.3399/BJGPO.2021.0020

**Published:** 2021-06-30

**Authors:** Paul Bogowicz, Helen J Curtis, Alex J Walker, Philip Cowen, John Geddes, Ben Goldacre

**Affiliations:** 1 Population Health Sciences Institute, Baddiley-Clark Building, Newcastle University, Newcastle upon Tyne, UK; 2 The DataLab, Nuffield Department of Primary Care Health Sciences, University of Oxford, Oxford, UK; 3 Department of Psychiatry, University of Oxford, Oxford, UK

**Keywords:** primary health care, antidepressive agents, quality of health care

## Abstract

**Background:**

Antidepressants are commonly prescribed. There are clear national guidelines in relation to treatment sequencing. This study examines trends and variation in antidepressant prescribing across English primary care.

**Aim:**

To examine trends and variation in antidepressant prescribing in England, with a focus on: monoamine oxidase inhibitors (MAOIs); paroxetine; and dosulepin and trimipramine.

**Design & setting:**

Retrospective longitudinal study using national and practice-level data on antidepressant items prescribed per year (1998–2018) and per month (2010–2019).

**Method:**

Class- and drug-specific proportions were calculated at national and practice levels. Descriptive statistics were generated, percentile charts and maps were plotted, and logistic regression analysis was conducted.

**Results:**

Antidepressant prescriptions more than tripled between 1998 and 2018, from 377 items per 1000 population to 1266 per 1000. MAOI prescribing fell substantially, from 0.7% of all antidepressant items in 1998 to 0.1% in 2018. There was marked variation between practices in past year prescribing of paroxetine (median practice proportion [MPP] = 1.7%, interdecile range [IDR] = 2.6%) and dosulepin (MPP = 0.7%, IDR = 1.8%), but less for trimipramine (MPP = 0%, IDR = 0.2%).

**Conclusion:**

Rapid growth and substantial variation in antidepressant prescribing behaviour was found between practices. The causes could be explored using mixed-methods research. Interventions to reduce prescribing of specific antidepressants, such as dosulepin, could include review prompts, alerts at the time of prescribing, and clinician feedback through tools like OpenPrescribing.net.

## How this fits in

The trend towards increased prescribing of antidepressants in English primary care is well documented. This study found evidence of continued rapid growth in prescribing, as well as substantial variation in prescribing behaviour between practices. Intervention is warranted to reduce prescribing of specific antidepressants such as dosulepin.

## Introduction

Antidepressants are commonly prescribed in English primary care, corresponding in 2017 to 6% of all drugs dispensed and costing £235 million (net ingredient cost).^1^  Antidepressants are categorised according to mechanism of action (Supplementary Table S1). There are four main classes: MAOI; tricyclic; selective-serotonin reuptake inhibitor (SSRI); and serotonin-norepinephrine reuptake inhibitor (SNRI). Other antidepressants are usually referred to as 'atypical'; this article uses 'other' in keeping with *British National Formulary* (BNF) terminology.

The National Institute for Health and Care Excellence (NICE) guideline for depression recommends the use of SSRIs over other antidepressants.^2^  The guideline notes that paroxetine has the highest incidence of discontinuation symptoms among SSRIs. The guideline recommends the use of an alternative SSRI or an 'other' antidepressant when switching owing to initial inadequate response.^2^  Less well-tolerated drugs, such as venlafaxine and tricyclic and MAOI antidepressants, feature later on in treatment sequencing. The guideline advises against the use of dosulepin. NHS England also advises against prescribing of trimipramine, in addition to dosulepin.^3^  Combinations of antidepressants may be used, although this would normally require input from a psychiatrist.^2^ 

OpenPrescribing.net (https://openprescribing.net) is a free online tool created by the research team to provide open access to longitudinal English primary care prescribing data at practice level. The authors set out to examine trends and variation in antidepressant prescribing in England, using data from OpenPrescribing.net and one other source. The focus is on prescribing of MAOIs as there are concerns about underuse of this class.^4^  Prescribing of paroxetine was also focused on, owing to concerns about discontinuation symptoms, and dosulepin and trimipramine, as guidelines advise against their use. This was a descriptive study and a hypothesis to test was not prespecified.

## Method

### Study design

A retrospective longitudinal study was carried out of antidepressant prescribing using routinely collected English primary care data. Aggregated annual prescription cost analysis (PCA) data and monthly practice-level prescribing data were used, which were available from OpenPrescribing.net. Both datasets record number of items of each drug preparation prescribed per year or month. An item is a measure of prescribing activity; each drug-specific item corresponds to a single prescription for that drug. Information on quantity (for example, number of tablets prescribed) is contained within the data, but the dosing regimen and course length cannot be accurately ascertained.

### PCA data

PCA data are freely available from NHS Digital.^5^  The data are stratified by year. Each dataset contains rows corresponding to all drugs in the *BNF*, by name and preparation or formulation. For each row, the total number of items dispensed in primary care in England is given. Longitudinal changes in nomenclature were accounted for by approximate matching where there were discrepancies between historical *BNF* codes and current codes. This process and other aspects of how PCA data were handled are described in detail elsewhere.^6,7^  All available data were extracted from 1998–2018 inclusive.

### Practice-level data

Practice-level summaries of prescribing activity are freely available from NHS Digital.^8^  This data is compiled by OpenPrescribing.net. The prescribing data are arranged by drug preparation name, month, and practice. NHS Digital organisation data were used to find practice setting and operational status.^9^  The searches were restricted to typical practices (setting code '4') in order to exclude atypical settings such as walk-in centres, out-of-hours services, and urgent and emergency care centres. All available monthly data were extracted, from August 2010 to November 2019 inclusive. For analysis of current prescribing, data were also extracted consisting of the last 12 months of prescribing activity (December 2018–November 2019), excluding dormant or closed practices.

### Data analysis

The *BNF* antidepressant categories were used as a basis for classification (Supplementary Table S1). Duloxetine and venlafaxine were removed from the 'other' category and were reclassified as SNRIs. Maprotiline, mianserin, and trazodone were removed from the tricyclic category and they were reclassified as 'other'. The rationale for this was that these drugs are chemically similar to drugs already in the 'other' group: mianserin to mirtazapine, for example.

#### PCA data

Stacked line charts were created after grouping by class, unadjusted and adjusted for mid-year population.^10^  Proportions corresponding to each class and for specific drugs were calculated by dividing the class or drug total by the overall total for that year. Line charts for proportions by class were obtained and for the 10 current most commonly prescribed antidepressants. Proportions for specific drug strengths were calculated by drug. Capsule and tablet forms were combined but not immediate release and extended or modified release. Specific drug strength line charts were obtained for the 10 current most commonly prescribed antidepressants.

#### Practice-level data

Proportions were calculated corresponding to each class and for specific drugs (as described for the PCA data), for each practice by month (2010–2019), and for the latest year. Percentile charts were created for the monthly data, by class and for specific drugs. Basic descriptive (summary) statistics were obtained for the latest year proportions, by class and for specific drugs. Heat maps were also created for the latest year for MAOI, paroxetine, dosulepin, and trimipramine prescribing, with practices grouped at clinical commissioning group (CCG) level.

Multilevel mixed-effects logistic regression analysis was carried out with prescribing of either dosulepin or trimipramine as dependent variable. The following categorical independent variables were included in the model:^11,12^  percentage proportion of patients aged >65 years; proportion of patients with a long-term health condition; practice list size; number of GPs per 1000 patients; rurality; Index of Multiple Deprivation (IMD); and Quality and Outcomes Framework (QOF) score. Unadjusted odds ratios (ORs) from univariate analyses were compared with adjusted ORs from an analysis, including all independent variables. Where variables were missing within the dataset, the corresponding practices were dropped from the analysis.

### Software and reproducibility

Data management was carried out using Google BigQuery and Python (version 3.6.5) running in Jupyter Notebook (version 5.5.0). Data analysis was carried out using Python and Stata ( version 13.1). Data and code used for management and analysis are freely available online (https://github.com/ebmdatalab/antidepressant-trends-paper/).

## Results

### PCA data

#### Overall trends

**Figure 1.**
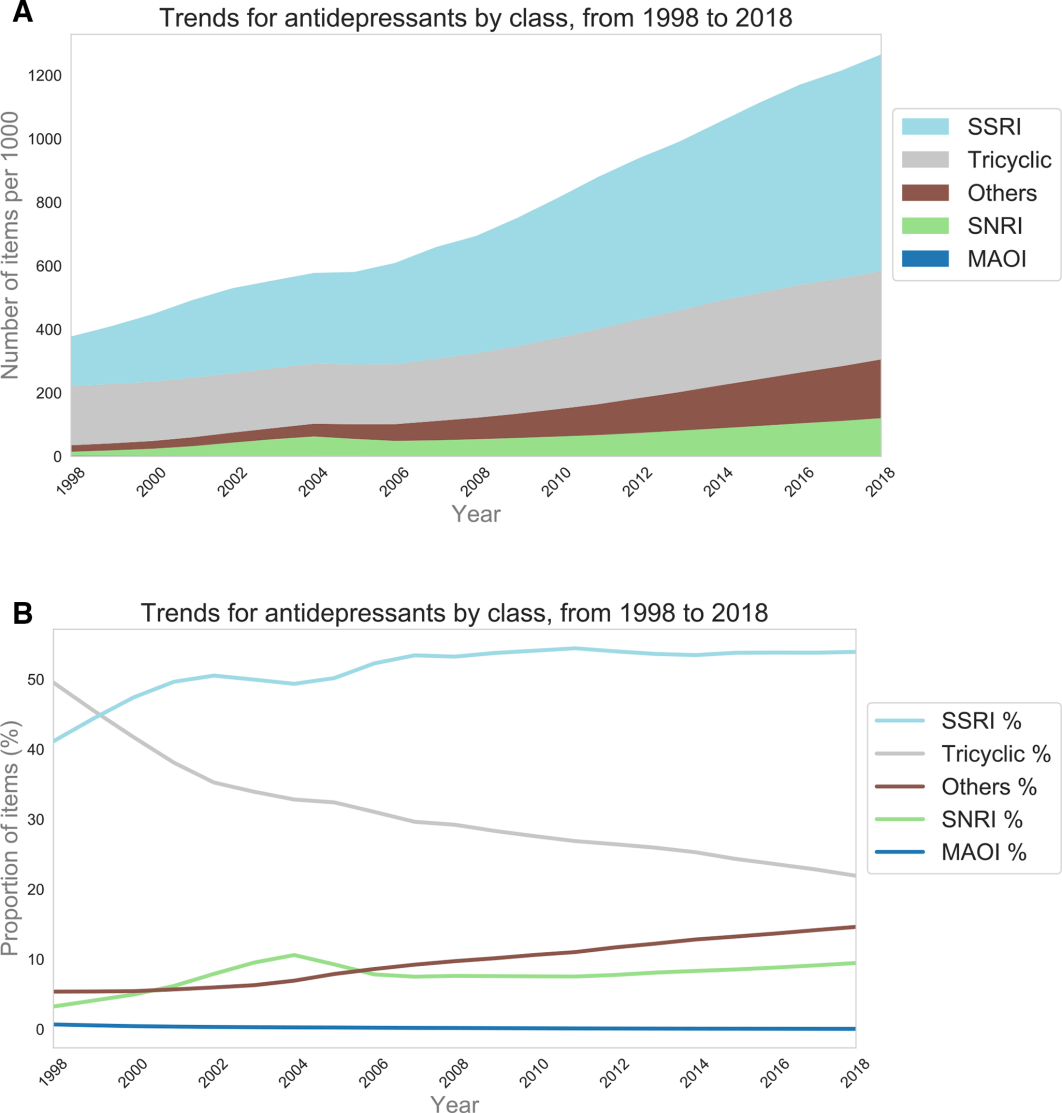
The population standardised numbers (A) and proportions (B) of prescribed antidepressant items by class, from 1998–2018. MAOI = monoamine oxidase inhibitor. SNRI = serotonin-norepinephrine reuptake inhibitor. SSRI = selective-serotonin reuptake inhibitor.

The number of antidepressant items prescribed per year more than tripled over the past two decades, from 18.4 million in 1998 to 70.9 million in 2018 (Supplementary Figure S1). The magnitude of change is the same even after accounting for population growth (Figure 1a). In 2018, the majority of prescribed antidepressant items were SSRIs (53.9%), followed by tricyclics (21.9%), 'other' antidepressants (14.6%), SNRIs (9.5%), and MAOIs (0.1%) (Figure 1b). This compares with 41.1%, 49.5%, 5.4%, 3.3%, and 0.7% in 1998, respectively.

**Figure 2.**
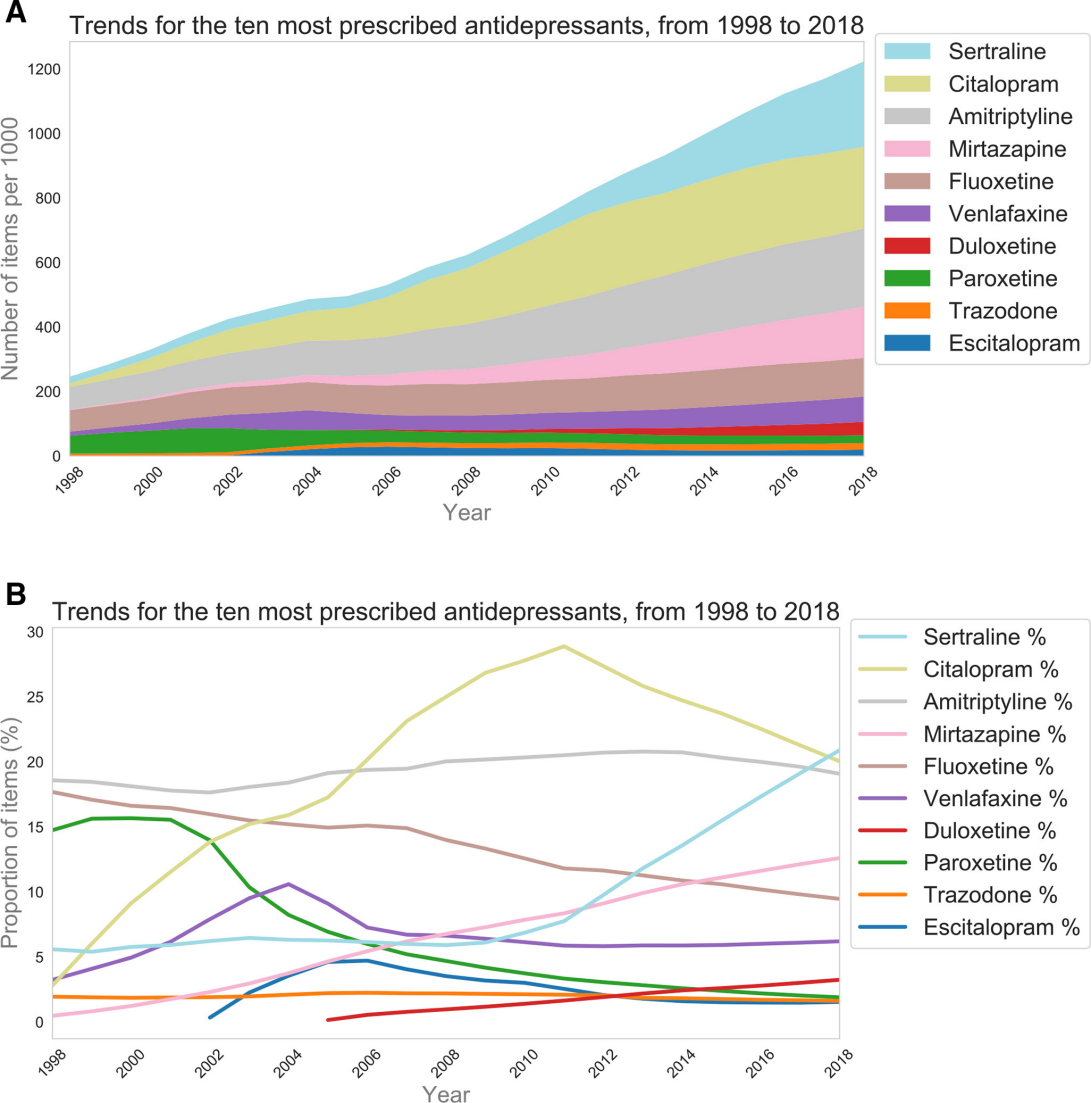
The population standardised numbers (A) and proportions (B) of prescribed antidepressant items for the 10 most commonly prescribed antidepressants (in 2018), from 1998–2018

The 10 current most commonly prescribed antidepressants accounted for 96.7% of all antidepressant items prescribed in 2018, but only 65.2% in 1998. Trends are shown in Figure 2. Trends were similar for unstandardised data (Supplementary Figure S2). Prescribing of citalopram increased markedly from 1998–2011, peaking at 28.9%. Prescribing of sertraline has increased markedly since 2012 (20.9% in 2018). Sertraline overtook citalopram as the most prescribed antidepressant in 2018. Prescribing of mirtazapine has been steadily increasing since 1998 (0.5% to 12.6% in 2018).

#### Prescribing of specific drug strengths

**Figure 3.**
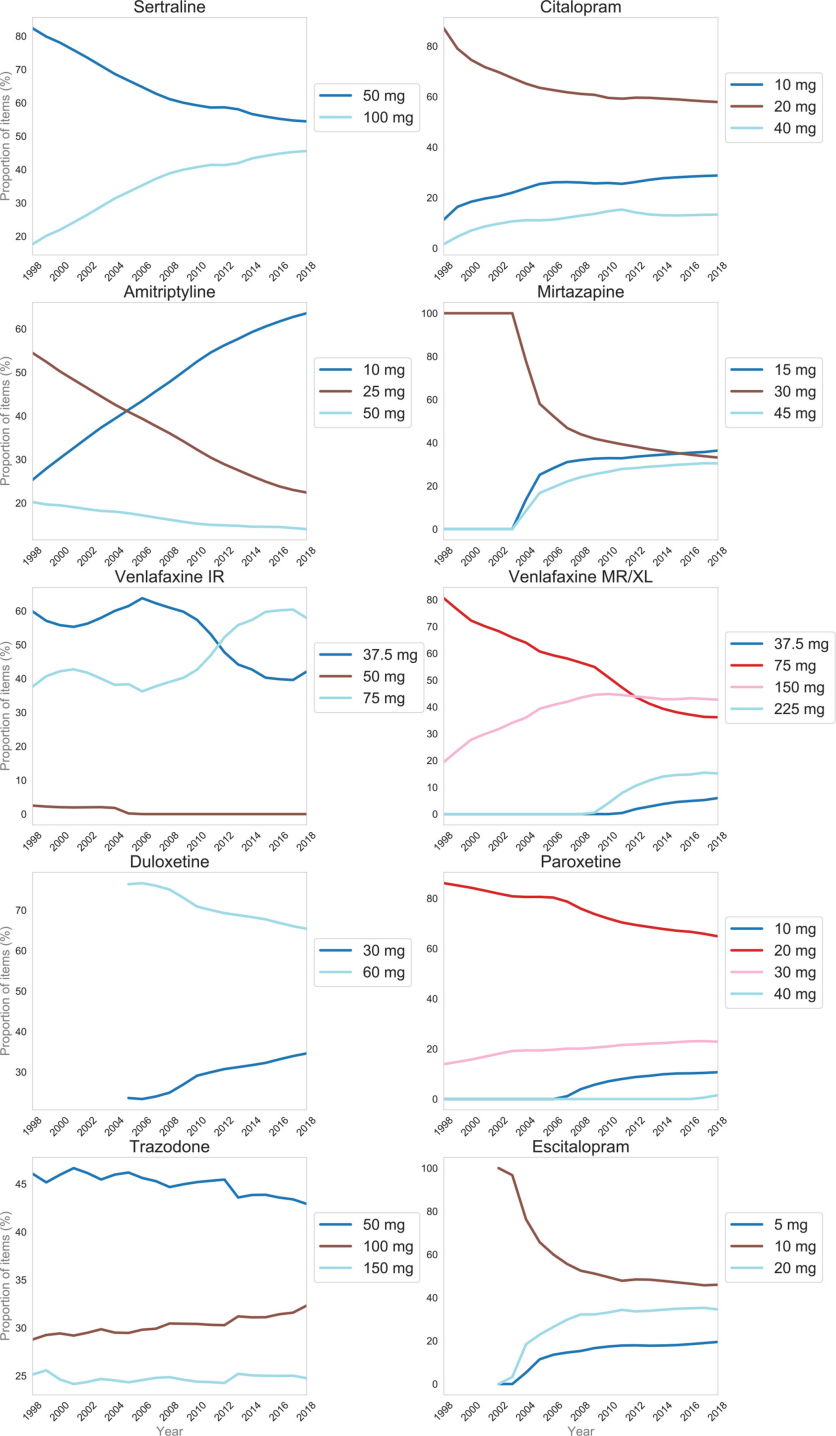
The proportions of specific antidepressant drug strengths prescribed by drug, for the 10 most commonly prescribed antidepressants (in 2018), from 1998–2018. Fluoxetine data not shown. IR = immediate release. MR = modified release. XL = extended release

Trends for specific drug strengths are shown in Figure 3. There is a trend towards increased prescribing of higher or highest strength forms for many drugs; for example, sertraline and mirtazapine. There has been a gradual but marked shift from prescribing higher strength forms of amitriptyline to the lowest strength form. Fluoxetine prescribing was static; the proportions accounted for by the 20mg forms ranged from 96% to 100% (data not shown).

### Practice-level data

#### Overall trends

**Figure 4.**
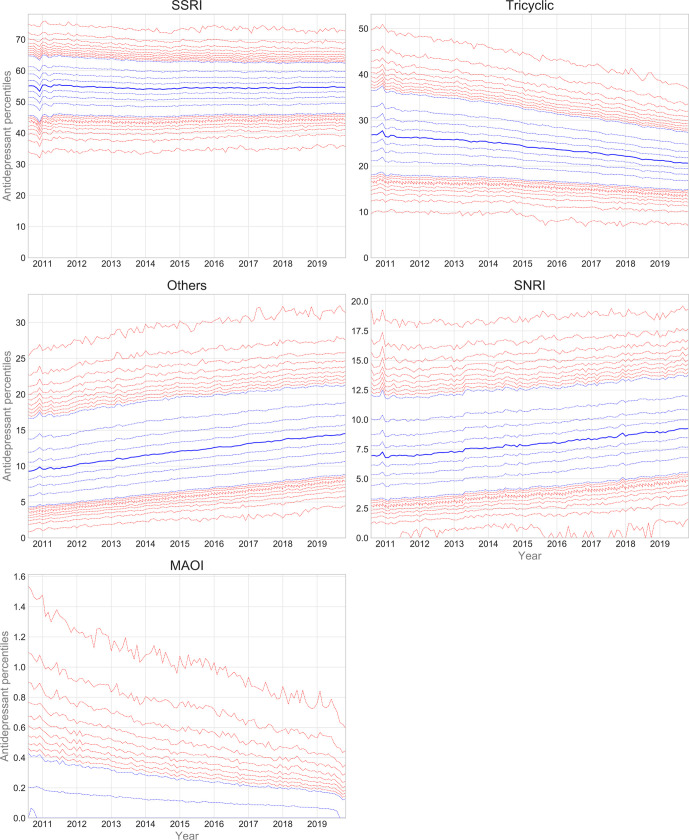
Practice-level percentile charts for the proportion of antidepressant items in each class, from August 2010–November 2019. Deciles are in blue, with median shown as a heavy blue line, and extreme percentiles in red. MAOI = monoamine oxidase inhibitor. SNRI = serotonin-norepinephrine reuptake inhibitor. SSRI = selective-serotonin reuptake inhibitor.

Trends and variation in the practice-level proportions of each class of antidepressants are presented in Figure 4. Supplementary Table S2 contains summary statistics for current (latest year) prescribing. There was marked variation in prescribing between practices, for all classes. For example, in the latest year, the MPP for SSRIs was 54.7%, with interdecile range [IDR] 15.8% (Supplementary Table S2). The corresponding charts and summary statistics for the 10 most prescribed drugs are presented in Supplementary Figure S3 and Supplementary Table S3, respectively.

#### Prescribing of MAOIs

Trends and variation in the practice-level proportions of individual MAOIs are presented in Supplementary Figure S4. Prescribing of all four drugs decreased over time. There was very little prescribing of isocarboxazid. Among the other drugs, prescribing appeared to be concentrated among a small proportion of practices (approximately 10% or fewer for each drug). This is reflected in the statistics for latest year prescribing (Supplementary Table S4). There was some variation in prescribing among the prescribing practices. Supplementary Figure S5 contains the heat map for CCG-level prescribing of MAOIs (pooled, given low numbers for individual drugs). There was variation in prescribing, with hotspots mainly in the South.

#### Prescribing of paroxetine, dosulepin, and trimipramine

**Figure 5.**
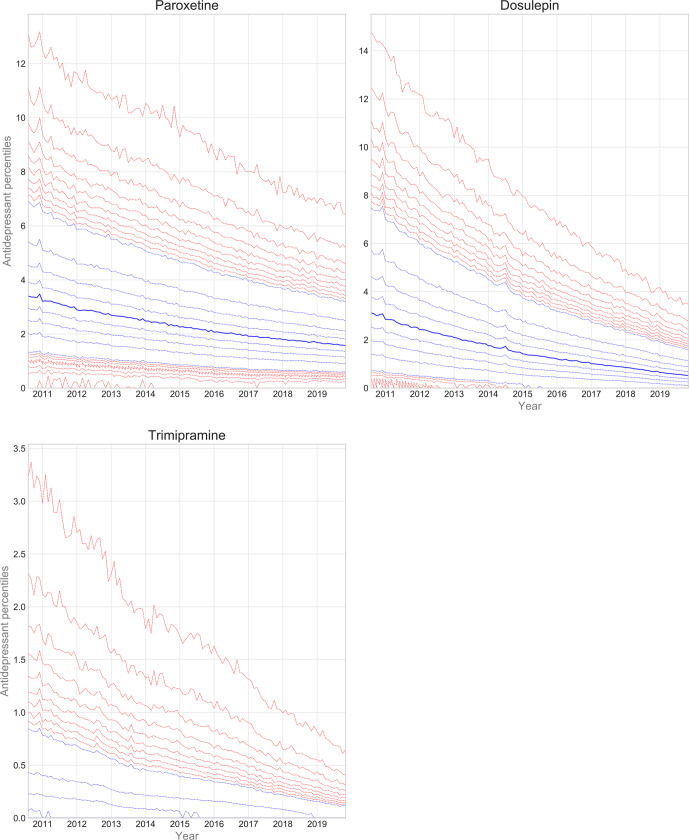
Practice-level percentile charts for the proportions of paroxetine, dosulepin, and trimipramine prescribed out of all antidepressant items, from August 2010–November 2019. Deciles are in blue, with median shown as a heavy blue line, and extreme percentiles are in red

Trends and variation in the practice-level proportions of paroxetine, dosulepin, and trimipramine are presented in Figure 5. Prescribing of all three drugs decreased over time. The spread of the deciles and extreme percentiles also decreased. Table 1 contains summary statistics for latest year practice-level proportions. There was marked variation in paroxetine (MPP = 1.7%, IDR = 2.6%) and dosulepin (MPP = 0.7%, IDR = 1.8%) prescribing. Variation in trimipramine prescribing (MPP = 0%, IDR = 0.2%) was confined to the upper deciles and extreme percentiles.

**Table 1.** Practice-level basic descriptive statistics for the proportions of paroxetine, dosulepin, and trimipramine prescribed between December 2018 and November 2019

	**Mean**	**Standard deviation**	**Lowest decile**	**Lower quartile**	**Median**	**Upper quartile**	**Highest decile**	**IQR**	**IDR**
Paroxetine %	1.9	1.3	0.7	1.2	1.7	2.4	3.3	1.3	2.6
Dosulepin %	0.9	0.9	0	0.3	0.7	1.2	1.9	0.9	1.8
Trimipramine %	0.1	0.2	0	0	0	0	0.2	0	0.2

IDR = interdecile range. IQR = interquartile range.

Supplementary Figure S6 contains the heat maps for CCG-level prescribing of paroxetine, dosulepin, and trimipramine. There was variation in prescribing of paroxetine and dosulepin, with hotspots scattered across the country. There was less variation in prescribing of trimipramine, with hotspots mainly in the South East.

Supplementary Table S5 contains the results of the logistic regression analysis. There was a moderate association between practice proportion of patients aged >65 years and prescribing of dosulepin and/or trimipramine. There were weak associations between practice IMD and QOF score and prescribing.

## Discussion

### Summary

The number of antidepressant items prescribed per year more than tripled over the past two decades. Prescribing of tricyclic antidepressants and MAOIs decreased, while prescribing of 'other' antidepressants increased (mirtazapine in particular). Prescribing of higher strength preparations increased for some drugs, suggestive of higher dose prescribing. There was significant variation in prescribing at practice level, by class and for specific drugs (paroxetine and dosulepin in particular).

### Strengths and limitations

A key strength of the study is that the data accounts for all English NHS primary care prescribing of antidepressants, rather than a sample. This is an advantage over the use of GP electronic health record databases, which currently do not have population-wide coverage. A second key strength of the study is the open-source nature of the data and code.

One limitation is the use of items prescribed as a measure of prescribing activity. The number of items prescribed for a particular patient may vary according to the prescriber and patient; for example, a 28-day supply could be prescribed as a single 28-day prescription (one item) or four seven-day prescriptions (four items). The number of items prescribed may also be greater than the number dispensed; the data only shows those dispensed. There is no comparable English dataset measuring an alternative to the item.

Antidepressant medication may be prescribed for other indications, such as anxiety disorders (especially SSRIs) and neuropathic pain (amitriptyline and duloxetine). The national prescribing datasets used do not contain information on indication. While electronic health record datasets can record the indication in principle, in reality these data are very incomplete. Where such data are recorded, antidepressant prescribing is typically associated with depression. One English study found that 61% of a small sample of those prescribed antidepressants had a diagnosis of depression or mixed depression and anxiety.^13^  In one small Scottish study, this figure was 85%.^14^  It is unclear why the figures differ. A more recent large UK GP database study reported that 67% had a diagnosis of depression.^15^  A review of the Read codes used suggests that this figure includes those with mixed depression and anxiety. The authors of the UK-wide study did not take indication into account in their primary analysis, possibly because this data is unreliable. The present study found that 64% of current (2018) amitriptyline prescribing is accounted for by the lowest strength (10 mg) forms. It can be inferred that the majority of current amitriptyline prescribing is low dose, and therefore for indications other than depression (recommended daily dose 100–150 mg).^16^ 

Data corresponding to private prescriptions and prescriptions issued in secondary care were not included in either of the datasets. However, the data should capture some secondary care prescribing, as many patients under the care of English community mental health services receive prescriptions from their primary care providers under shared care arrangements.

### Comparison with existing literature

It was found that the number of antidepressant items prescribed per year more than tripled over the past two decades. This is consistent with the findings of a recent UK GP database data study, which included data for 1995–2011.^15^  The authors of a previous English PCA data study, which included data for 1998–2010, suggested that increasing antidepressant prescribing may be related to population growth, use of antidepressants for non-depressive indications, and longer-term prescribing.^17^  Two UK GP database data studies found that increases were mainly owing to the latter.^15,18^  A Scottish study suggested that changes were owing to increasing numbers of patients being prescribed antidepressants and use of higher doses, in addition to longer-term prescribing.^19^  A trend towards longer-term prescribing has also been noted elsewhere in the world.^20,21^  Longer-term prescribing may be because of, in part, difficulties stopping antidepressants,^17^  and the perceived safety of SSRIs for long-term treatment when compared with older antidepressants. Longer-term prescribing is also considered to be standard practice for prevention of relapse in recurrent depression.^22^  Increasing primary care prescribing of antidepressants is likely to also reflect a shift towards shared care arrangements, with a corresponding decrease in secondary care prescribing.

A decreasing trend was found in MAOI prescribing. This may reflect a lack of knowledge and experience of prescribing these drugs, particularly among recently trained psychiatrists.^4^  Doctors may also be reluctant to prescribe MAOIs because of concerns about food–drug and drug–drug interactions.^4^  A decreasing trend was also found in citalopram prescribing, although it remains the second most prescribed antidepressant. This trend may be related to the 2011 and 2014 warnings about the potential for citalopram to cause QT interval prolongation,^23,24^  as well as the 2011 recommendation from NICE to use sertraline for generalised anxiety disorder.^25^  An increasing trend in mirtazapine prescribing was found. This may reflect its appeal as an alternative for those with contraindications to or adverse effects from SSRIs and SNRIs. Mirtazapine is also often combined with SSRIs to augment their effect; however, this may be of little benefit in primary care populations.^26^ 

Evidence was found of significant variation in prescribing at practice level, by class, and for specific drugs such as dosulepin. It is unclear why this variation exists. Some may be warranted, accounted for by case-mix and patient preference. Some of the variation is almost certainly unwarranted. It was found that in November 2019, dosulepin accounted for over 3% of antidepressant prescribing for some outlying practices. NICE guidelines have advised against the use of dosulepin since at least 2009.^27^  It is unclear whether the differences between practices are owing to variation in incident prescribing or in approaches to tackling legacy prescribing. The variation may also reflect underlying differences in local-level psychiatric service provision, in relation to starting and/or reviewing use of drugs such as dosulepin. There may be other drivers of unwarranted variation more generally, including the use of local formularies, financial pressures, and the influence of industry. A recent Scottish qualitative study found that GP prescribing of antidepressants is influenced by GP anxiety in relation to recurrence of depression, having few concerns about drug safety, and a lack of routine medication reviews.^28^ 

### Implications for research and practice

The drivers of the variation in practice-level prescribing could be explored using a mixed-methods approach, using data to target a range of prescribing behaviours. In particular, further research could explore GPs’ knowledge of antidepressant guidelines and attitudes towards legacy prescribing. Intervention to reduce variation could include prompts to review, alerts at the time of prescribing, and automated monthly reporting on extreme or unusual prescribing. A recent position statement from the UK Royal College of Psychiatrists has called for the use of technology in tracking and reviewing long-term antidepressant prescribing.^29^  The authors note that a free practice-level monthly monitoring service is available through OpenPrescribing.net for any drug, including dosulepin.
